# Enhancement of aerodynamic performance of axial compressors utilizing natural aspiration through optimized casing circumferential slot

**DOI:** 10.1038/s41598-024-64659-4

**Published:** 2024-06-14

**Authors:** Peyman Ghashghaie Nejad, Reza Taghavi Zenouz

**Affiliations:** Aerodynamic and Compressible Turbomachine Research Laboratory, School of Mechanical Engineering, Iran University of Science and Technology (IUST), NarmakTehran, 16846-13114 Iran

**Keywords:** Axial compressor, Stall margin, Natural aspiration, Tip leakage flow, Engineering, Aerospace engineering, Mechanical engineering

## Abstract

This paper presents results of aerodynamic enhancement of axial compressors utilizing optimum natural aspiration through a circumferential slot made within the casing wall upstream the rotor blades row. The method of investigation is based on numerical simulation of flow field. Geometries of the slot walls were optimized to find the maximum stall margin and pressure ratio of the compressor. The optimum case is accompanied by boosting more momentum within the blades tip region through the slot. Consequently, tip leakage vortex flow weakens and compressor stability margin increases. Final results showed that in comparison to the untreated casing, the optimum geometry of the slot causes the total pressure ratio and stall margin to increase by 4.2% and 3%, respectively. This state-of-the-art technique is simple and economic which can be easily implemented in practical cases while the compressor is exposed to commencement of flow instabilities in the forms of the rotating stall or surge phenomena.

## Introduction

Modern compressors of gas turbine engines, utilized in aircraft or other applications, are regularly developed to increase their efficiency and to achieve higher total pressure ratios with less degradation of their performance^1^. This is usually accompanied by increasing the stage loading which causes the engine weight and length to decrease^2^. Increased stage loading frequently triggers amplification of flow instabilities in the form of rotating stall or surge^3^. Thus, appropriate stability enhancement methods should be utilized especially when the stall margin is not adequate to ensure trustworthy operation^4^.

Flow structure near the rotor blades tip region has dominant effect on the flow stability^5,6^. Flow instability in its different types of rotating stall or surge is highly affected by the tip leakage flow structure^7,8^. A promising and validated approach is to employ casing treatments in its various types^9,10^. Amongst many different types of casing treatment can be referred to circumferential grooves^11,12^, axial slots^13^ and air injection^14,15^.

Rotor blades tip leakage flow is substantially influenced by the boundary layer structure formed on the endwalls^16^. Generally speaking, many passive and active methods are developed to control the boundary layer formed over solid walls of the objects exposed to the fluid flow, so far^17,18^. These methods are widely focused on controlling the onset of transition from laminar to turbulent flow or to delay the boundary layer separation point^19^. Flow suction or blowing through the solid walls are amongst the most conventional methods in this respect^19,20^. This method has been extensively used in both the internal and external flows such as in turbomachines and aircraft exterior surfaces.

Cao et al.^21^ have described flow mechanism and aspiration strategies in an ultra-highly loaded supersonic axial compressor. Through their numerical investigations they concluded that, via appropriate mass flow suction from the endwall, the corner separation can be effectively removed. Merchant^22^ numerically studied influence of boundary layer suction on turbine exit guide vanes and on two stages of an axial compressor. He concluded that high loading of the blades could be obtained along the blade span with only a relatively small amount of aspiration.

A three-stage counter rotating compressor was examined by Freedman^23^ to study effects of aspiration on the surfaces of the blades as well as on the end walls. He used this method to maintain the boundary layer attached to the solid walls under high loading conditions, in order to decrease the losses. Dang et al.^24^ numerically modeled aspirated compressor blades employing 3D inverse approach. They qualified the blade design process utilizing full interaction between a specified transpiration scheme and prescribed pressure loading. Their results indicated that an optimum aspiration can minimize the suction rate needed for efficiency enhancement at design and off-design conditions. Jain et al.^25^ through their numerical simulations evaluated impact of aspiration slots arrangement on the efficiency and stall margin of a transonic axial compressor rotor blades.

Generally speaking, blades tip leakage flow can significantly affect aerodynamic performance of any dynamic turbomachine. Therefore, it would be important to use suitable techniques to control and alleviate its undesirable aerodynamic effects. An effective method is air injection at the blades tip region. This can be performed using an external source, for example, compressed air supplied by another compression system^26^. In fact, using this technique causes the fluid particles momentum to increase in the blades tip region. Consequently, the trajectory and strength of the tip vortex flow can be controlled in order to meet the best performance. Boosting the air flow in the blades tip region can be performed naturally, i.e., without utilizing any external sources.

In the previous study of the present authors^27^, the challenges associated with blades tip leakage flow in axial compressors were addressed through the exploration of three distinct strategies: circumferential tape, S-shape nozzle and natural aspiration slot, all imposed upstream the rotor blades row. Aerdynamics of each of the above-mentioned technique was compared with results of the untreated case. Notably, the natural aspiration slot overcome the other proposed methods. In comparison to the untreated case, the natural aspiration approach demonstrated a remarkable increase of 3.5% in the total pressure ratio and a surge margin improvement of 3%. Moreover, the mass flow rate through the blades row tip gap exhibited a notable increase of 2.33 g/s.

In the present research work aerodynamic performance of a low-speed isolated rotor blades row of an axial compressor is enhanced utilizing natural aspiration of air through a circumferential slot made within the casing wall upstream the blades. The method of investigation is based on numerical solution of the governing equations. Performance curves and flow fields are obtained and discussed under different flow conditions and slot geometries. These results helped in to find the best geometry of the slot to maximize the compressor pressure ratio and stall margin via numerical optimization process.

## Model specifications

**Figure 1:**
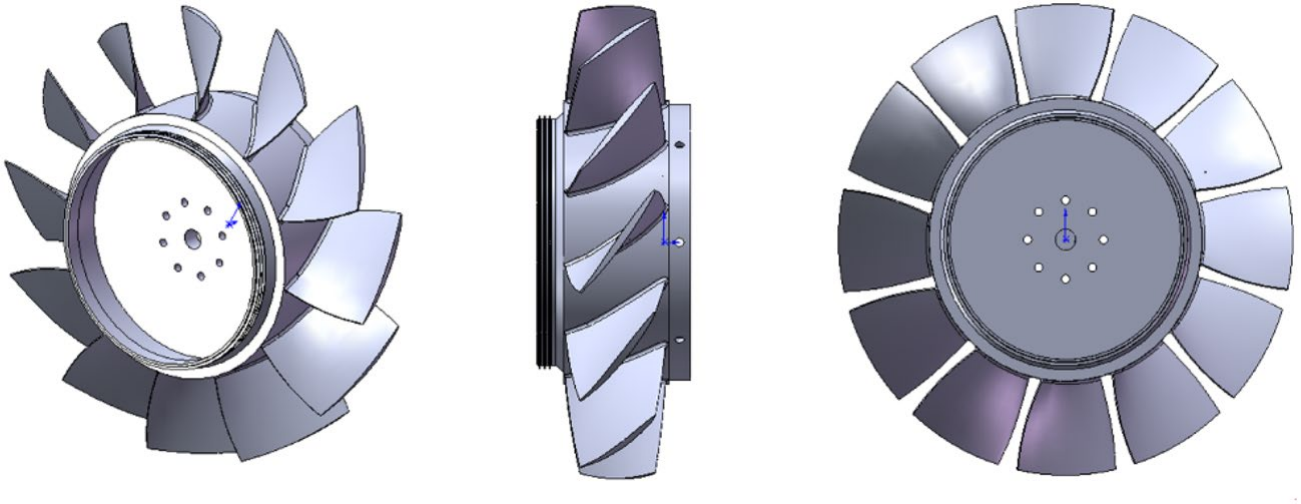
Different views of rotor blades row.

An isolated rotor blades row of an axil compressor is considered for the present study. It includes 12 blades, each having a radial cross section geometry based on NACA-65 series airfoils. Figure 1 shows different views of this rotor blades row and Table 1 lists its specifications. This rotor has already been tested experimentally by Inoue^28^.

**Table 1:** Rotor blades row specifications.

**Parameter**	Value	Unit
Rotational speed (N)	2000	r/min
Hub diameter (D_h_)	270	mm
Hub-to-tip ratio (D_h_/D_t_)	0.6	–
Tip clearance to blade chord ratio (t/C_t_)	1.7	%
Tip chord length (C_t_)	117.5	mm
Blades tip solidity (σ_t)_	1	–
Blades tip stagger angle (γ)	56.2	deg

**Figure 2:**
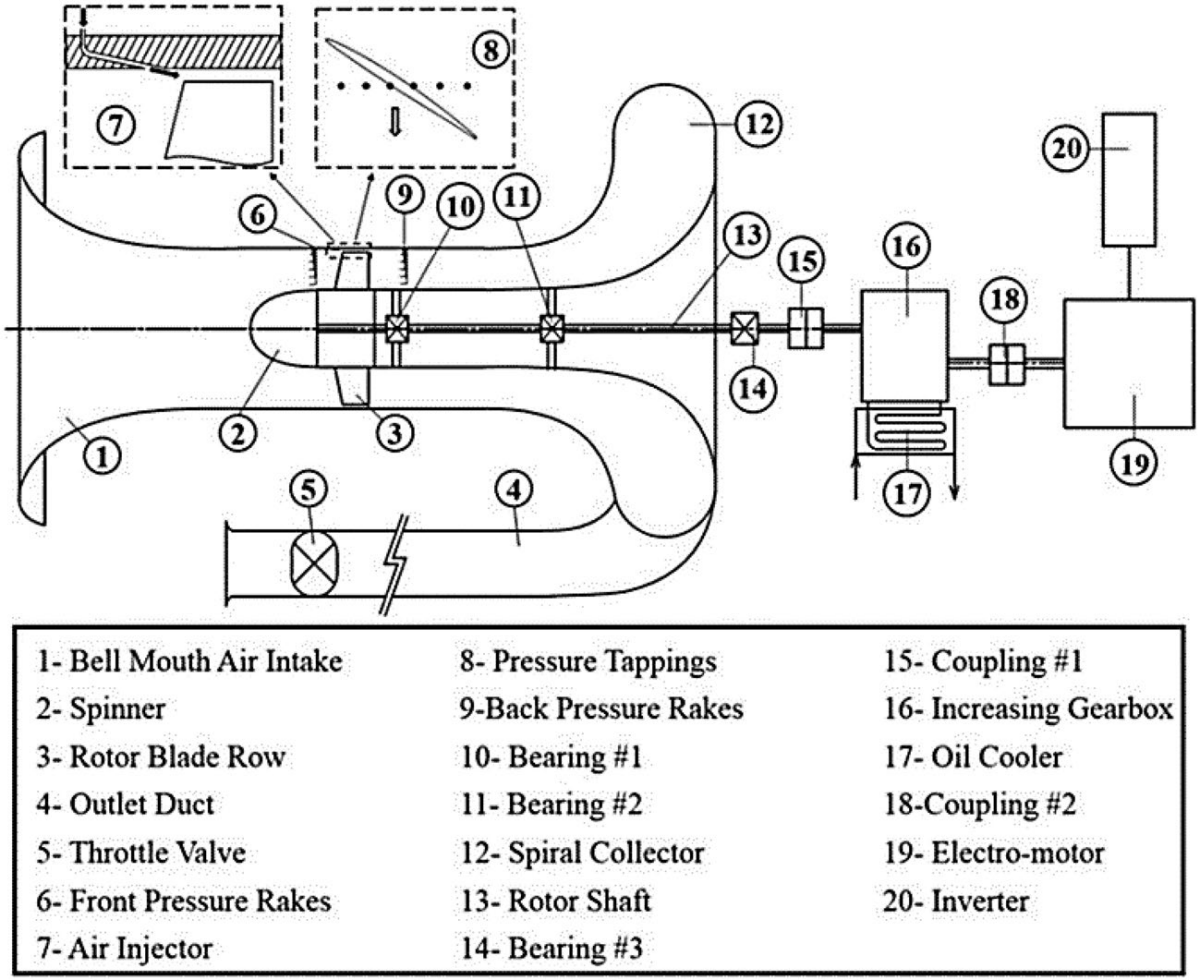
Layout of IUST-ACTLab low-speed axial compressor test-rig.

Taghavi et al.^29^ have already conducted many tests on this rotor blades row in a low speed axial compressor test-rig located at the Aerodynamic and Compressible Turbomachine Research Laboratory of the Iran University of Science and Technology (IUST-ACTLab). The layout of this test-rig is shown in Fig. 2. The compressor axis was driven by an electro motor and its speed was controlled via an inverter. A throttle valve, positioned downstream of the rotor blade row, was used for adjusting the main mass flow rate. A proper number of pressure tappings and hot-wire probes were used for logging the time averaged and instantaneous pressures and flow velocities. They were radially and circumferentially distributed at entry and exit regions of the rotor blades row.

**Figure 3:**
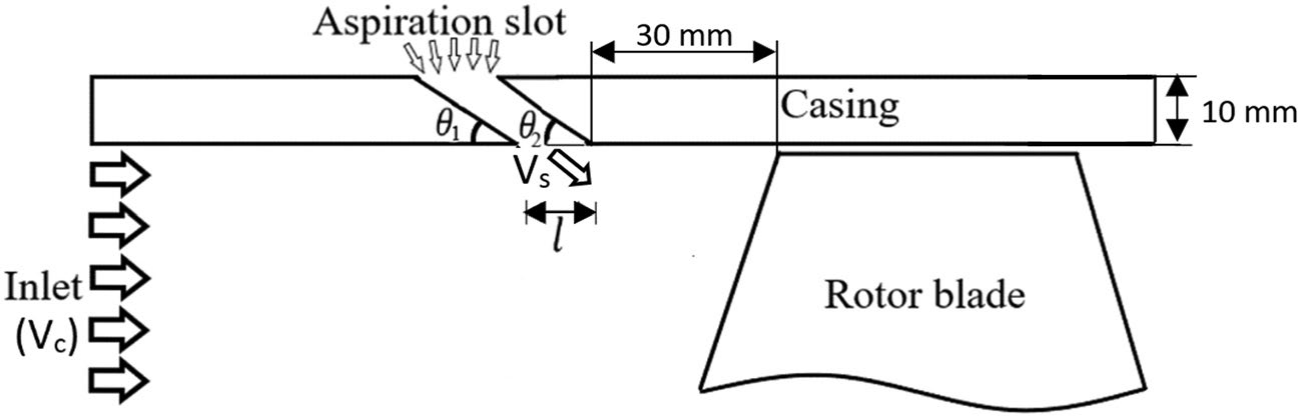
Schematic drawing of meridional view of rotor blade and aspiration slot.

A circumferential slot is made within the compressor casing wall upstream the rotor blades row. While the compressor is running the air flow is naturally being sucked into the flow passage. As will be shown in Section "Results and discussion", the slot geometry is optimized somehow to provide the best flow pattern in the blades tip region. As a result, aerodynamic performance of the rotor blades row would be augmented via using this state-of- the-art technique. Figure 3 schematically shows meridional view of the rotor blade and the aspiration slot. For a constant casing wall thickness the parameters of ${\theta }_{1}, {\theta }_{2}$ and $l$ (designated in Fig. 3) can fix the slot geometry.

## Numerical flow simulation technique and validation

One quarter of the model, including three blades, is considered in the present study. To simulate the turbulent flow, the well-known Shear Stress Transport (SST) k–ω model is utilized in ANSYS-CFX software. This model, proposed by Menter^30^, is a two-equation eddy-viscosity model which is widely used in separated flows by many researchers.

The governing main equations are introduced below through Eqs. (1), (2), (3).

### Continuity equation

1 $$\frac{\partial \rho }{\partial t}+\nabla .\left(\rho .\overrightarrow{u}\right)=0$$

### Momentum equations

Momentum equations for rotating sub-domains of the main solution field are the Navier–Stokes equations in rotating frame. The vector form of these equations is presented by the following equation.

2 $$\frac{\partial \overrightarrow{u}}{\partial t}+\overrightarrow{u}.\nabla \overrightarrow{u}=\frac{-1}{\rho }\nabla P-\overrightarrow{\Omega }\times \left(\overrightarrow{\Omega }\times \overrightarrow{r}\right)-2\overrightarrow{\Omega }\times \overrightarrow{u}+\nu {\nabla }^{2}\overrightarrow{u}$$

Second and third terms on the right hand side of the above equation are centrifugal and $\text{Coriolis}$ accelerations, respectively.

### Energy equation

The energy conservation of a fluid particle is ensured by equating the rate of total energy change to the sum of the net rate of the work done on the fluid particle, net rate of heat transfer to the fluid and the rate of increase of energy due to different sources. So, the total energy equation can be introduced by the following equation.

3 $$\rho \frac{DE}{Dt}=div\left(\rho u\right)+\left[\frac{\partial (u{\tau }_{xx})}{\partial x}+\frac{\partial (u{\tau }_{yx})}{\partial y}+\frac{\partial (u{\tau }_{zx})}{\partial z}+\frac{\partial (v{\tau }_{xy})}{\partial x}+\frac{\partial (v{\tau }_{yy})}{\partial y}+\frac{\partial (v{\tau }_{zy})}{\partial z}+\frac{\partial (w{\tau }_{xz})}{\partial x}+\frac{\partial (w{\tau }_{yz})}{\partial y}+\frac{\partial (w{\tau }_{zz})}{\partial z}\right]+div\left(k\; grad\; T\right)+{S}_{E}"$$

In the above equations,$u, x, t,$ $\rho$, $E$, $T$, ${S}_{E}$ and $\tau$ are velocity, distance, time, density, energy, temperature, specific energy and shear stress, respectively.

The commercial software of ANSYS-CFX was used for mesh generation within the computational domain. The grids for the circumferential slot were selected as hexahedral type, which is advantageous for maintaining high-quality grids, especially in regions with complex geometries. In addition, connection of the slot grids to the rotor casing was established through careful implementation of the frozen rotor boundary condition. This type of boundary serves as an interface between the rotating and stationary regions, providing coherent simulation of the flow field around the rotor blades and the circumferential slots.

**Figure 4:**
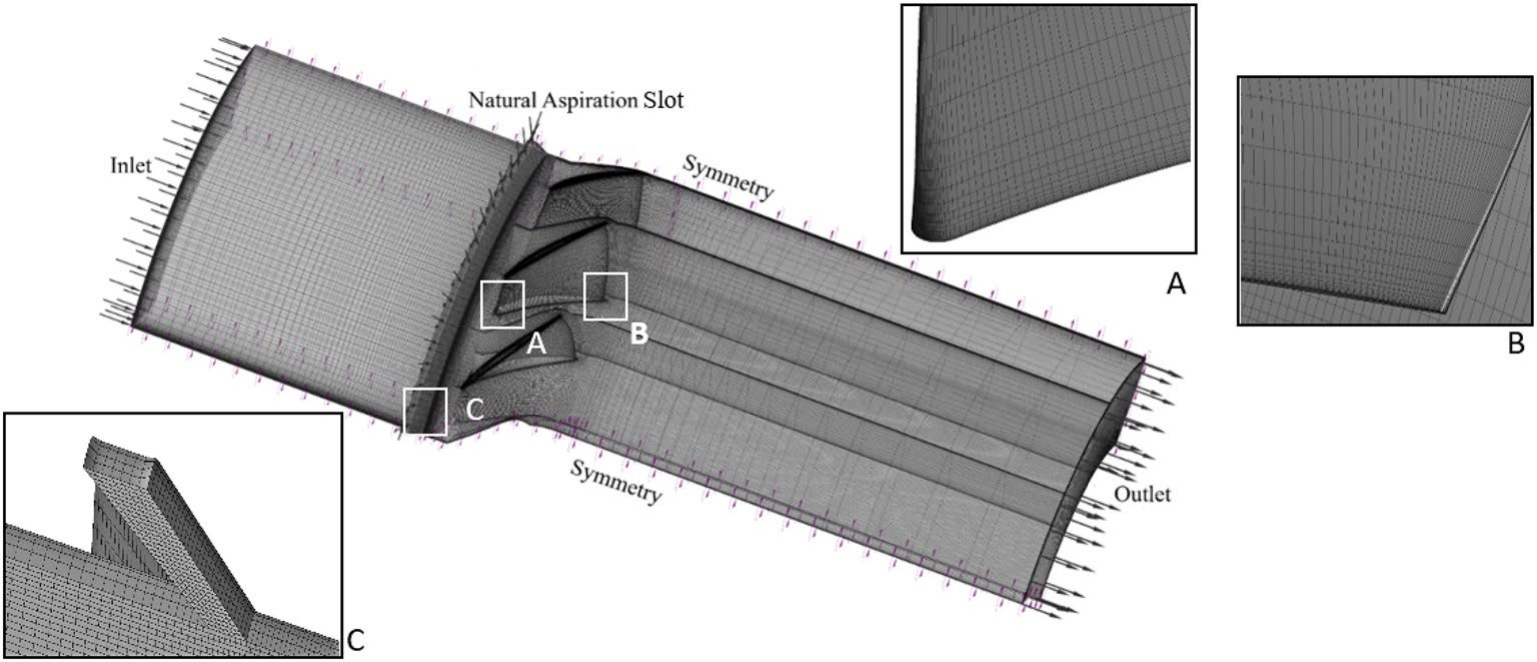
Surface grid structure.

The SIMPLE algorithm was used to solve the pressure–velocity coupling to enforce mass conservation and to obtain the pressure field. Figure 4 shows distribution of the grids generated on the solid walls. It is crucial to accurately capture the near-wall flow phenomena. So, the y + was kept less than 5.2, which causes the mesh to be likely well-resolved within the boundary layer. The mesh predominantly consists of hexahedral elements. These elements are advantageous for maintaining the high-quality grids, especially in regions with complex geometries. The mesh structure in the regions of high sensitivity, like interface of the slot and casing and also the leading and trailing edges of the blade, are zoomed out and shown in this figure.

To accurately model the interface between the slot and the blade passage mesh structures, the general connection model is combined with a frozen rotor approach. This method ensures that the data transfer between the slot outlet and the casing inlet is handled efficiently, maintaining the integrity of the flow characteristics. The general connection model allows for a uniform integration of different mesh regions. While the frozen rotor approach provides a steady-state approximation of the relative motion between the rotor and the stationary components. This approach ensures that the simulation procedure accurately captures the interactions and fluid dynamics near the interface.

Total pressure and temperature and flow angle are fixed on the inlet boundary. Non-slip and adiabatic conditions are imposed on the solid walls. The relative static pressure on the outlet boundary is gradually increased from zero to the value necessary for the onset of stall phenomenon. The frozen rotor boundary condition is considered for the interface between the rotating and stationery regions. Periodic boundary conditions are imposed on the lateral surfaces of the solution domain. Table 2 summarizes the boundary conditions used in the present study.

**Table 2:** Boundary conditions.

Inlet boundary	Outlet boundary
Total pressure: 101 kPa	Static pressure: 101 kPa-300 kPa
Total temperature: 288.15 K	

**Figure 5:**
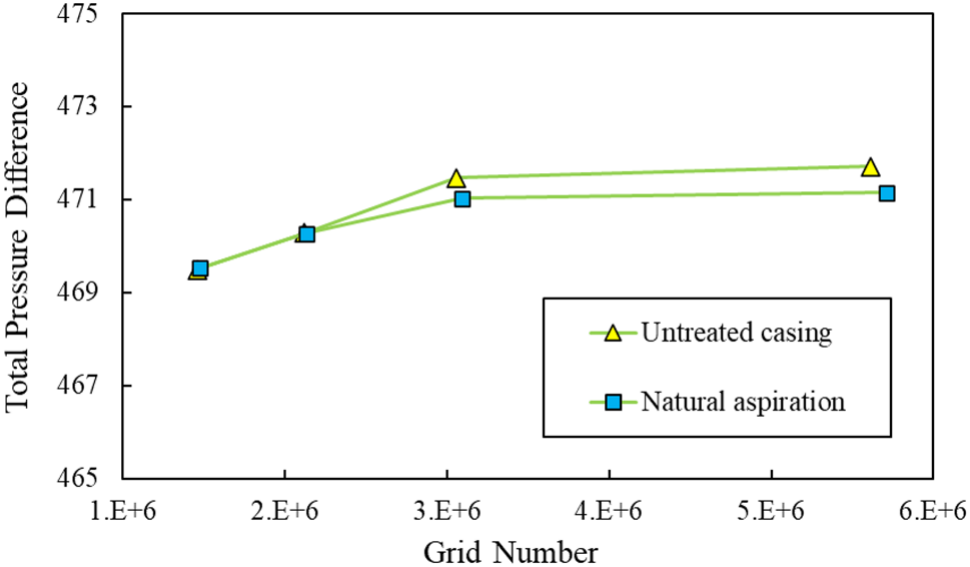
Results of mesh independency studies.

Figure 5 presents results of mesh independency studies for two cases of the untreated casing and in the presence of the circumferential slot. The plot depicts variations of the average total pressure difference versus the number of grids. As can be detected from this figure the total mesh count of approximately 3,100,000 is appropriate. In other words, no significant variation in the pressure difference can be observed beyond this latter value.

The geometry of the slot already introduced in the previous section (see Fig. 2) is numerically optimized via computational fluid dynamic technique. In this respect, the well-known Renormalization Group (RNG) k-$\upvarepsilon$ model was utilized in ANSYS-FLUENT to model the turbulent flow. Generally speaking, this latter model is assumed to correct the deficiency of the ordinary k–ε model in determining the turbulence kinetic energy^31^. The objective functions through the optimization process are the average velocities of the mainstream (V_c_) and exit flow from the slot (V_s_), which should be maximized. No flow separation occurs upstream the blades. So, using (RNG) k-$\upvarepsilon$ model seemed appropriate, due to its rapid convergency in comparison to the (SST) k–ω model. It was necessary to run so many cases in order to train the neural network required for the optimization process. More details about optimization process are presented in Section “Optimization Process”.

**Figure 6:**
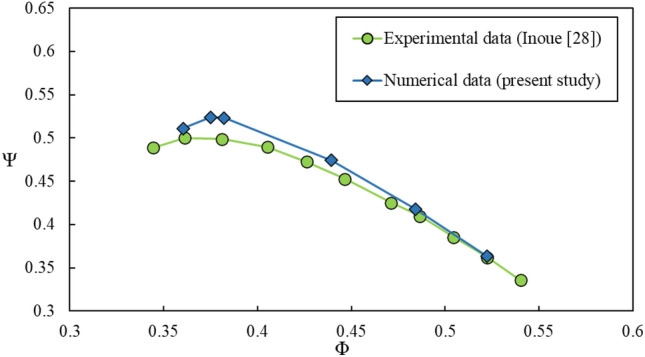
Rotor blade row performance curve.

Performance curve of the model, in terms of variations of the total pressure rise coefficient $\left(\psi =\Delta P/0.5\rho {U}^{2}\right)$ versus the flow coefficient $\left(\phi ={C}_{a}/U\right),$ is shown in Fig. 6. *∆p* is the pressure difference across the compressor. Present numerical result is compared with experimental result of Inoue et al.^28^. The maximum discrepancy is about 4.8%, which is satisfactory.

## Results and discussion

As already mentioned in Section "Model specifications", initially the slot geometry is optimized to obtain the best aerodynamic performance of the rotor blades row. Then, the flow field is simulated in detail for various blades tip clearances for the optimized and untreated cases.

### Optimization process

The main target of optimization process is to obtain the compressor maximum pressure ratio and stall margin. These will be attained if the air velocities at the compressor entry (${V}_{c}$) and slot exit (${V}_{s}$) to reach their maximum values through optimization of the slot geometry (${\theta }_{1}, {\theta }_{2}$ and $l$). Therefore, ${V}_{c} \text{and} {V}_{s}$ are considered as the objective functions and ${\theta }_{1}, {\theta }_{2}$ and $l$ are set as the cost functions.

**Figure 7:**
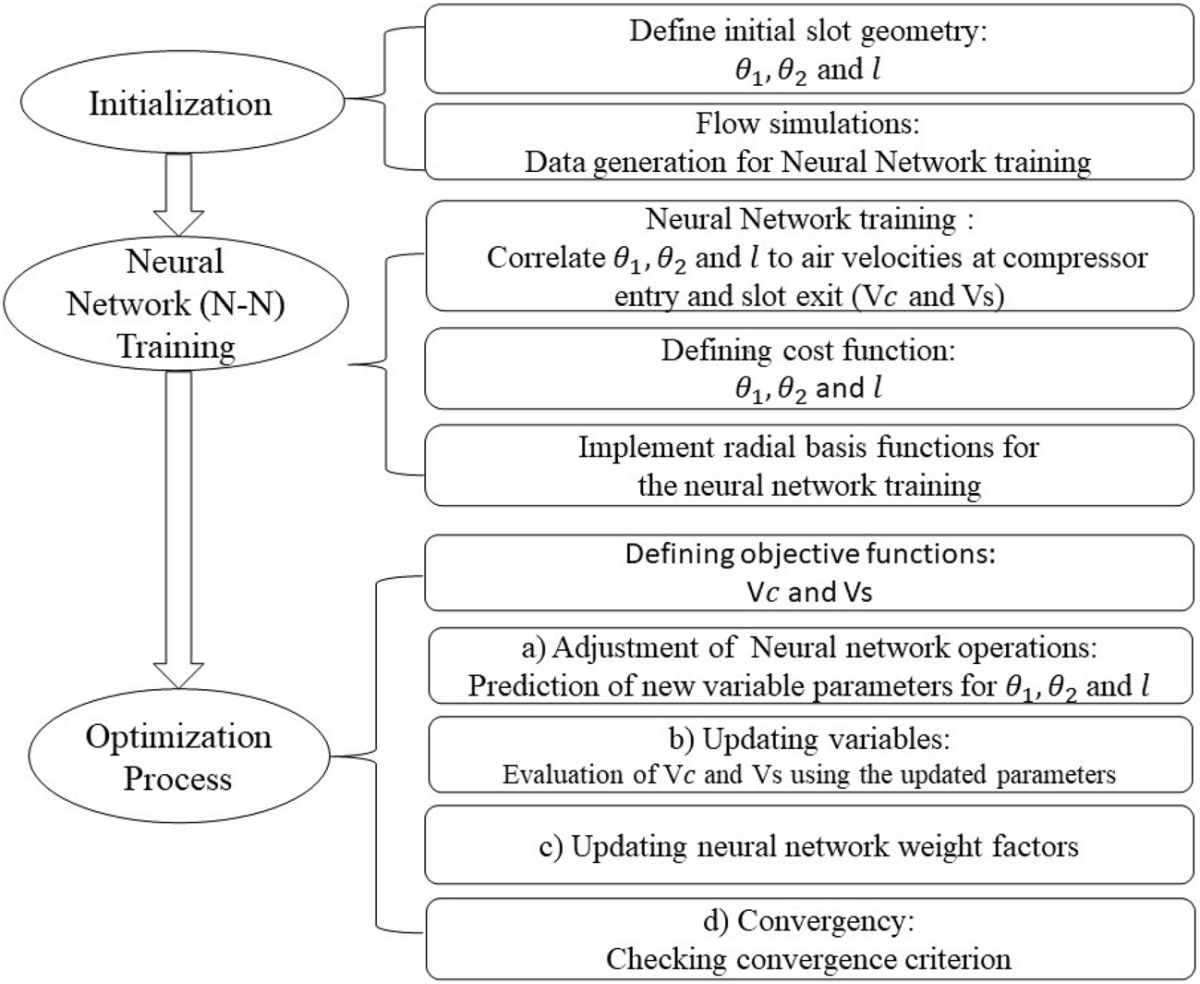
Optimization flow-chart.

Figure 7 introduces the optimization flow-chart. Numerous three-dimensional viscous flow simulations were executed to obtain data needed for training the neural network process, prior to embarking on the optimization phase based on the genetic algorithm.

The neural network architecture employed for the optimization purpose consisted of the radial basis functions layers, with two neurons in each layer. Comprehensive dataset was assembled for training the neural network, incorporating a range of slot dimensions (${\theta }_{1}, {\theta }_{2}$ and $l$) within the specified limits. The dataset included various operating conditions and corresponding performance metrics to facilitate robust training and subsequent optimization. Generally speaking, the radial basis functions are commonly used for approximations, interpolations, and optimizations which require complex functions. They are favored for their computational simplicity and ability to generalize to higher-dimensional spaces.

The optimization process entailed a meticulous examination of 46 configurations, including 45 cases for different slot dimensions (5 cases for each of the ${\theta }_{1}, {\theta }_{2}$ and $l$ parameters) and one case representing the plane case (in the absence of slot). The iterative adjustments made during the optimization process, guided by the neural network and radial basis functions, contributed to this comprehensive dataset (i.e., 46 dataset). This approach ensured a thorough exploration of the design space, capturing the influence of different slot configurations on the optimization of the objective function. The optimization process adhered to the specified constraints for the slot dimensions. *l* ranged from 4 to 20 mm and θ_1_ and θ_2_ ranged from 20 to 30 degrees. The parametres ${V}_{c} \text{and} {V}_{s}$ together with their relevant weighting factors served as the guiding metric for the neural network to iteratively adjust the slot parameters towards the optimal values. The convergence criterion through the optimization process was based on using the following correlation. The expression ${J}^{(k)}$ represents the value of the objective function at the kth iteration. $\epsilon$ is a negligible threshold (set equal to ${10}^{-6}$) determining the allowable changes in the objective function value.

$$\frac{\left|{J}^{(k)}-{J}^{(k-1)}\right|}{{J}^{\left(k-1\right)}}\le \epsilon$$

Design parameters are the aspiration slot dimensions (${\theta }_{1}, {\theta }_{2}$ and $l$). In other words, the slot geometry plays a crucial role in assessing its impact on the fluid dynamic of the blade tip leakage flow. It can enforce direct effect on the average velocities at the slot exit (Vs) and compressor entry (Vc). These parameters serve as variables through the optimization process. Their best configuration will provide the best aerodynamic performance of the rotor blades row. In other words, the optimum slot geometry will cause the blade tip leakage vortex strength to weaken.

**Figure 8:**
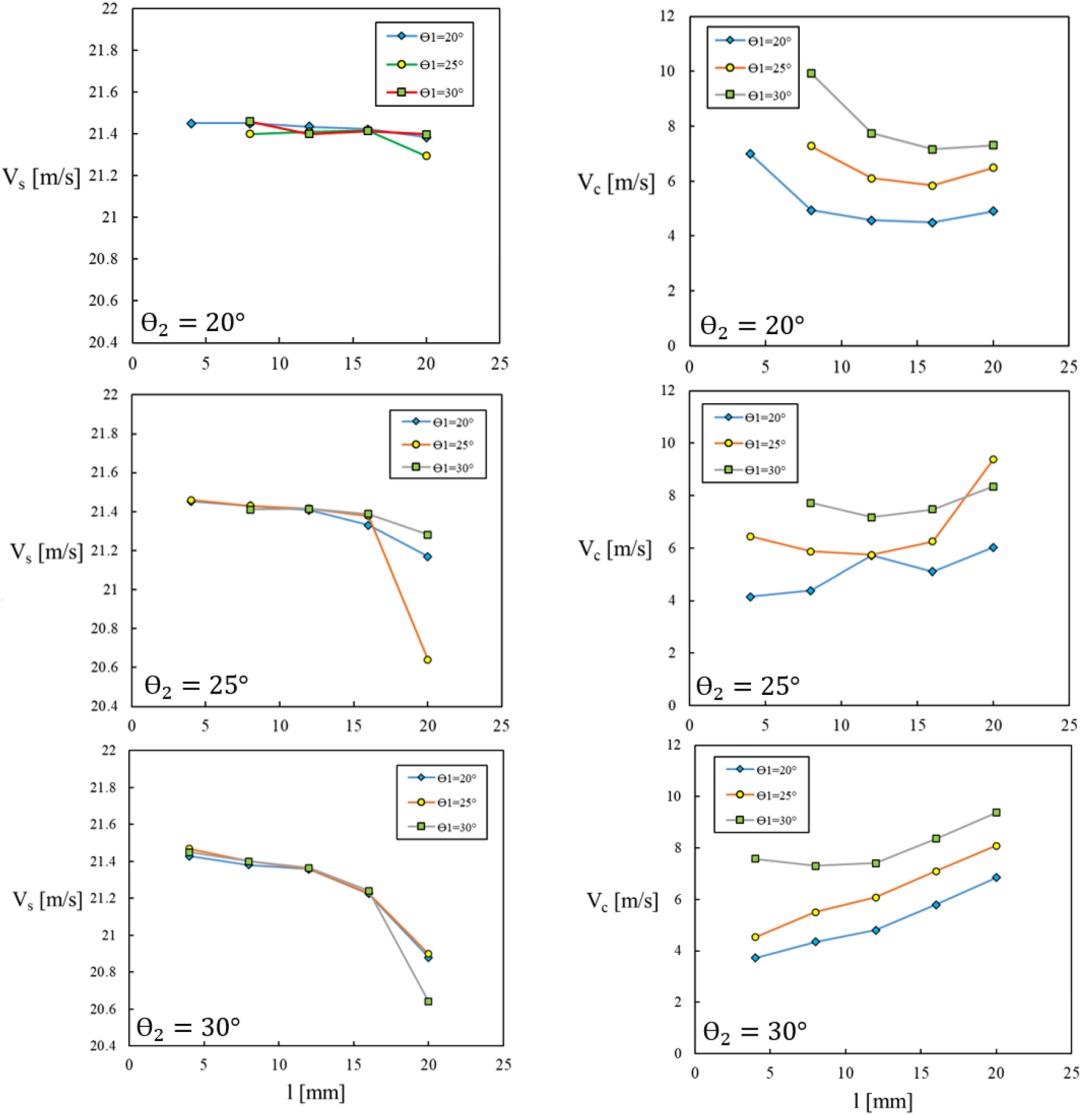
Effects of slot geometry on average velocities at slot exit and compressor inlet.

Among numerous results some of them are presented in Fig. 8, in terms of the variations of V $c$ and Vs versus the slot exit length (*l*) at various slot wall inclination angles (${\theta }_{1}$ and ${\theta }_{2}$).

Executing the optimization process provided to obtain the best values for the aspiration slot dimensions, as: *l* = *4.1 mm, *${\theta }_{1}=20.04^\circ$* and* ${\theta }_{2}=25.10^\circ$. These values were rounded to *l* = *4 mm, *${\theta }_{1}=20^\circ$* and* ${\theta }_{2}=25^\circ$, because the differences between the latter values with those of the former showed negligible effects on the flow field results.

### Performance curves

**Figure 9:**
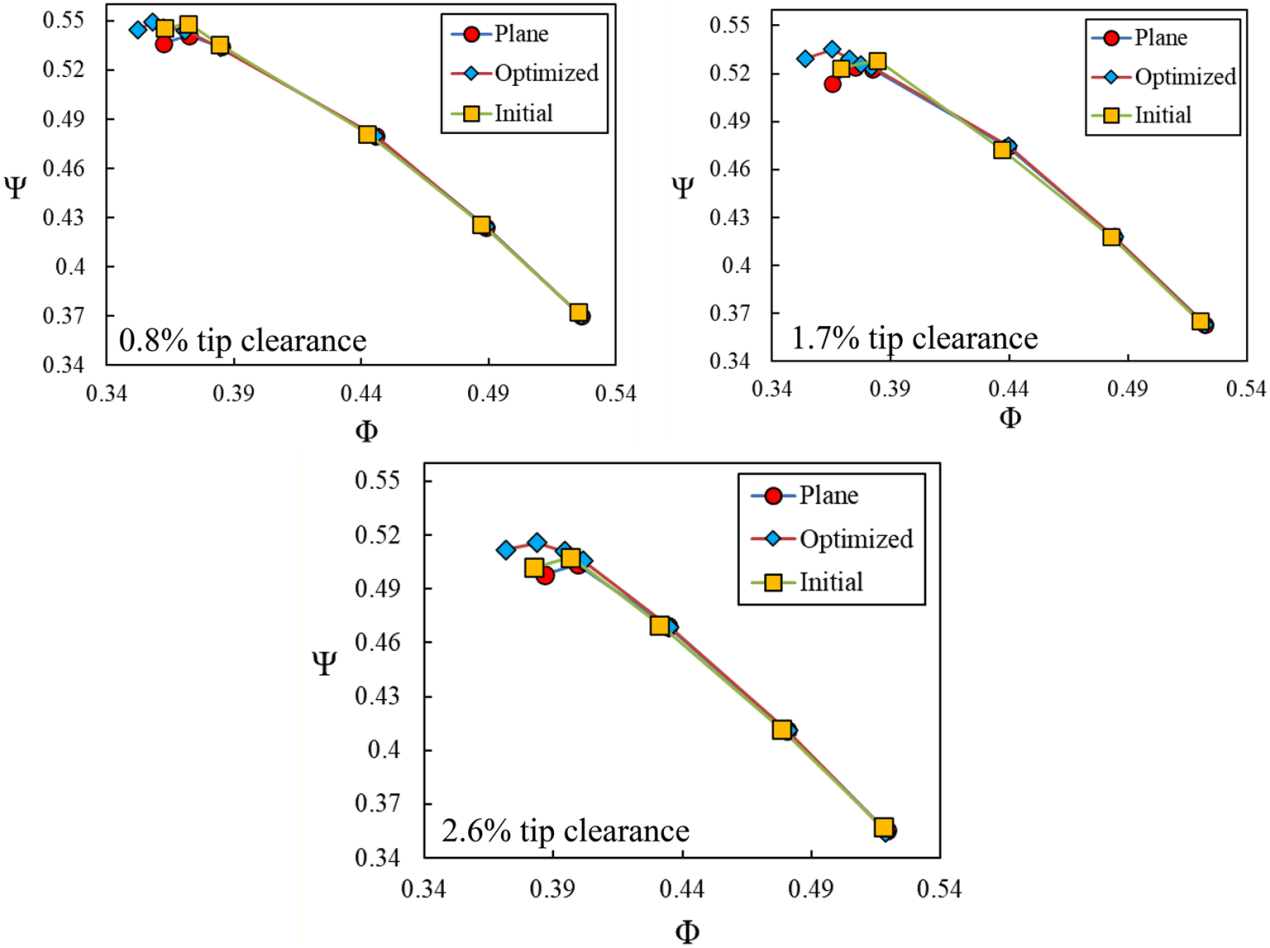
Performance curves for various cases.

Effectiveness of natural aspiration and its slot geometry was checked by investigation on the rotor blades row performance curves, in terms of variations of the pressure rise coefficient with the flow coefficient. Figure 9 presents these results. Three curves are designated in this figure as; “plane”: referring to the untreated casing, “initial”: referring to the initial or unoptimized slot and finally “optimized”: referring to the optimized slot. Results are presented for three tip clearance sizes of 8, 1.7 and 2.6% of the blade tip chord length. It should be noted that the slot geometry with *l* = *2 cm,* ${\theta }_{1}=15^\circ$ and ${\theta }_{2}=15^\circ$ was considered as the first guess (initial case) for commencement of the optimization process.

Based on the results presented in Fig. 9, the optimum case is accompanied by more pressure rise coefficients and wider stall margins for all the three blades tip clearance sizes. For example, at 1.7% blades tip clearance, the total pressure ratio and stall margin are increased by 4.2% and 3%, respectively. Improvement in stall commencement point for the optimum aspiration case in comparison to the untreated (or plane) case can be quantified by using the following correlation.

$$\overline{\Delta \varphi }=\frac{{\varphi }_{NA}-{\varphi }_{PL}}{{\varphi }_{PL}}$$

In the above correlation, ${\varphi }_{NA}$ and ${\varphi }_{PL}$ refer to the stall flow coefficients in the natural aspiration and the plane cases, respectively. Table 3 summarizes $\overline{\Delta \varphi }$ values for different blades tip clearances. The mass flow rate through the middle blade tip clearance is calculated and results are introduced in Table 3. It can be deduced that the optimum case has caused the blades tip flow rate to increase for all the blades tip clearance sizes.

**Table 3:** Stall onset improvement of natural aspiration case in comparison to untreated case for various blades tip clearances.

Blades tip clearance (%)	Stall onset improvement ($\overline{\Delta \varphi }$)	Blade tip clearance mass flow rate (g/s)	
		Plane case	Optimized aspiration case
0.8	2.7 %	3.7	3.8
1.7	3.0 %	7.5	7.7
2.6	3.9 %	12.1	12.3

### Flow structure

**Figure 10:**
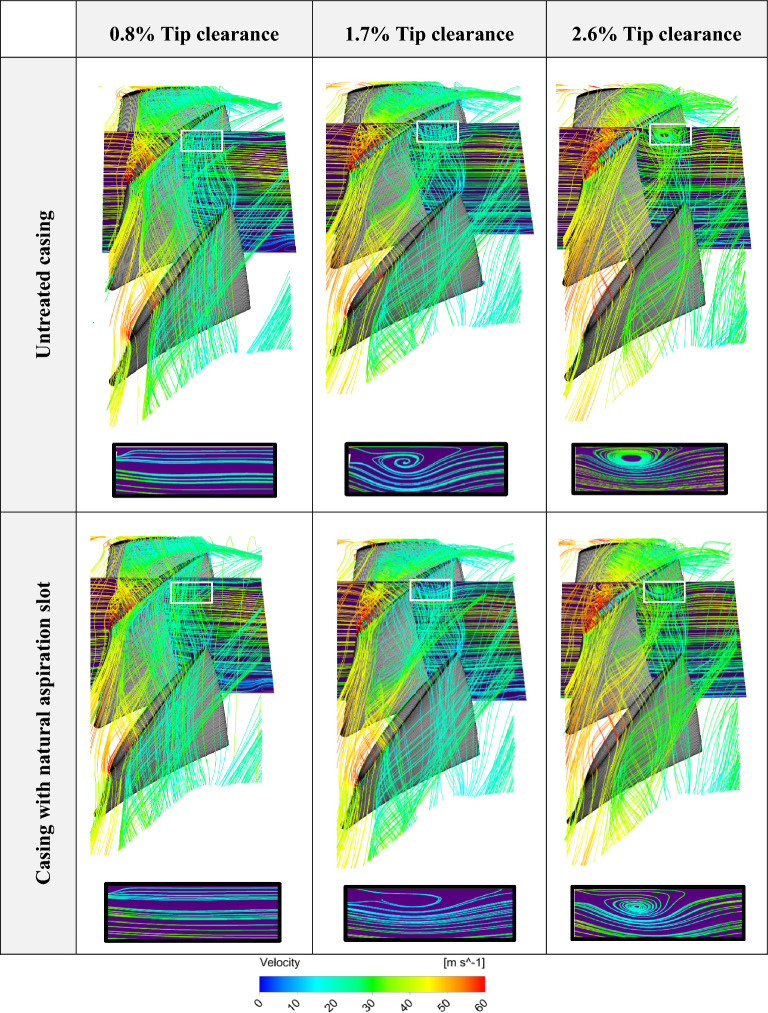
Flow pattern for untreated and treated compressor casings.

Flow patterns for untreated casing and the case in the presence of the circumferential natural aspiration slot is presented in Fig. 10 for the three blades tip clearances. For a better illustration of the beneficial effects of utilizing the natural aspiration technique, the projection of the streamlines on an imaginary rectangular window, set along the axial direction (meridional plane), is carried out and results are zoomed out and shown below each flow pattern. Focusing on these results one can easily deduce that the blades tip vortex flow has weakened while applying the natural aspiration in comparison to the untreated case.

**Figure 11:**
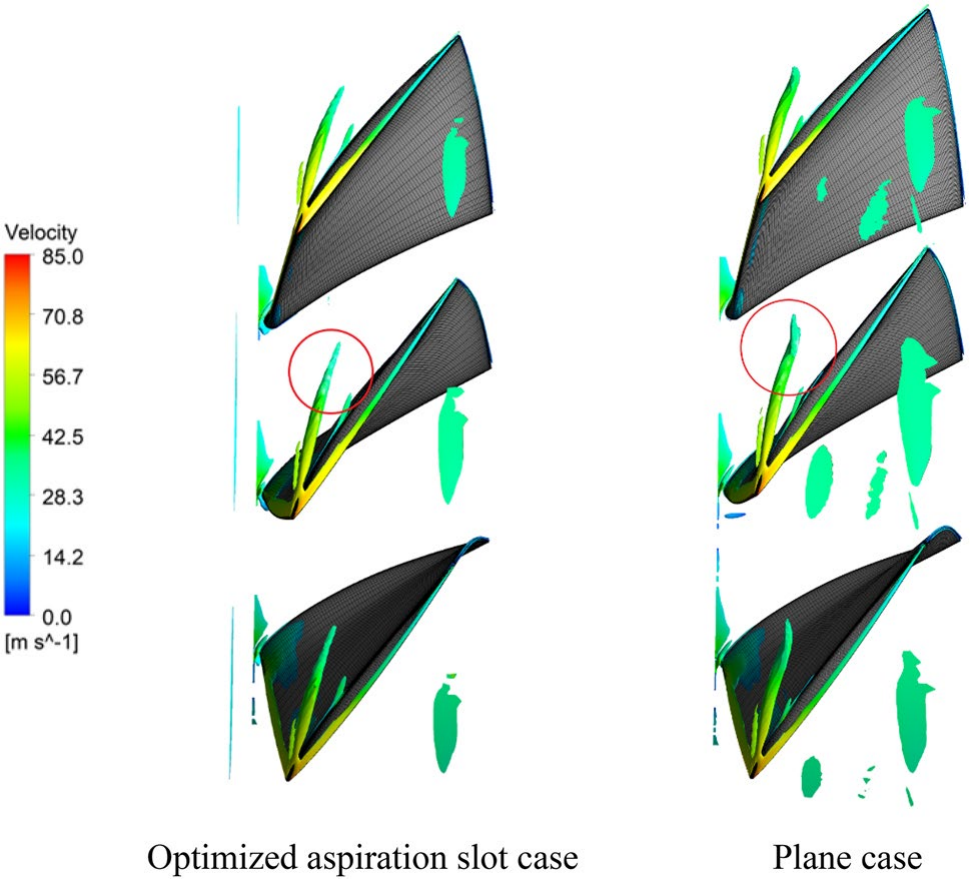
Tip vortex structure (Q = 2.5 × 10^7^ s^−2^) for optimized aspiration slot and plane cases with 1.7% blade tip clearance.

Flow field, especially in turbomachines, can be characterized by focusing on rather high-turbulent vortical regions. The well-known Q-criterion can be utilized to capture the vortices formed at the blades tip region. This latter criterion reflects both the vorticity and strain rate of the fluid elements. Q-criterion of *2.5* × *10*^*7*^* s*^*−2*^ was selected to display vortex structure for 1.7% blade tip clearance. These results are presented in Fig. 11 for the plane (without slot) and the optimized aspiration slot cases. It can be detected from this figure that the blade tip vortex, formed in the plane case, tends to lean towards the compressor circumferential direction (see the region inside the red circle). This orientation imposes blockage to the flow at the blade tip region, which is not desirable. It can be observed in this figure that aspiration through the slot has caused the tip vortex trajectory to direct towards the axial direction, thereby mitigating the flow blockage.

**Figure 12:**
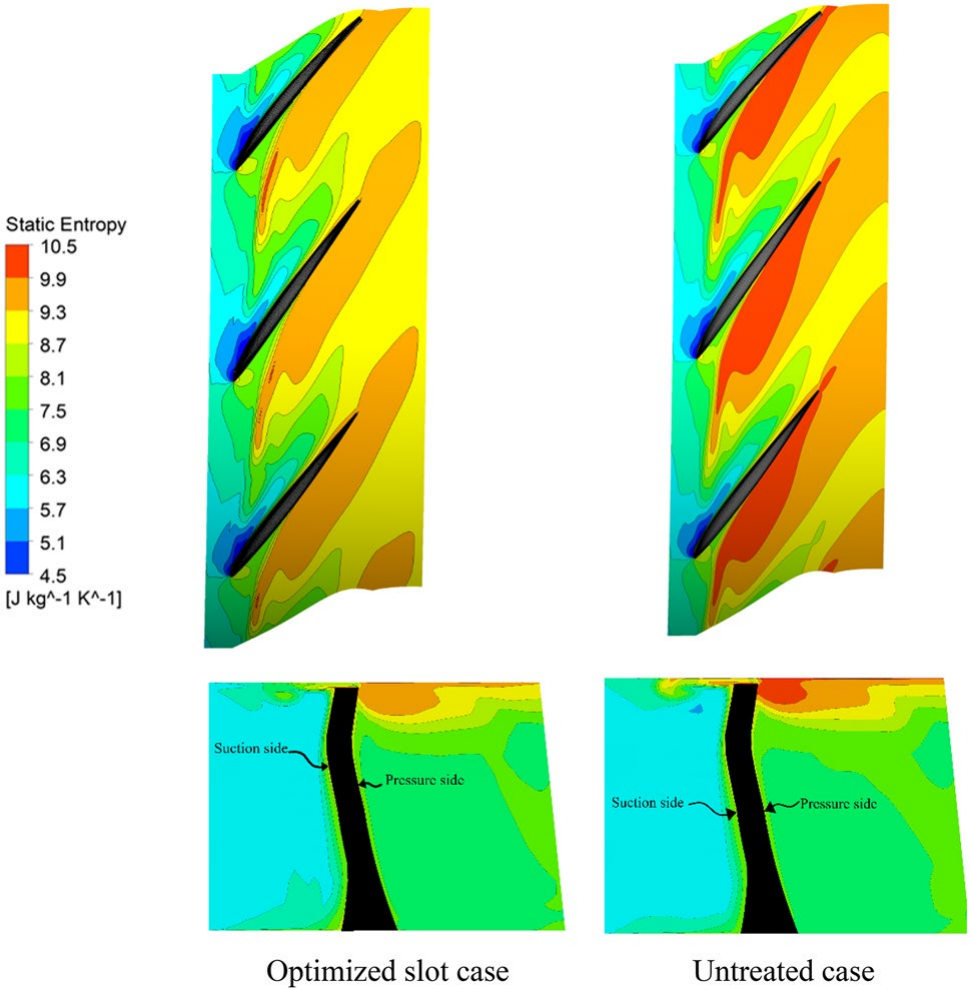
Entropy contours on 95% blade span and on an axial plane cutting the blade at its mid-chord, for optimized slot geometry and plane case with 1.7% blade tip clearance.

Figure 12 shows entropy contours at 95% blades span and on an axial plane cutting the blade at its mid-chord, for the optimized aspiration slot geometry and untreated cases for 1.7% blade tip clearance. It can be observed that the entropy intensity in the optimized case is lower in comparison to the untreated case, indicating beneficial effect of utilizing natural aspiration technique, due to its capability in alleviation of the blade tip leakage strength.

**Figure 13:**
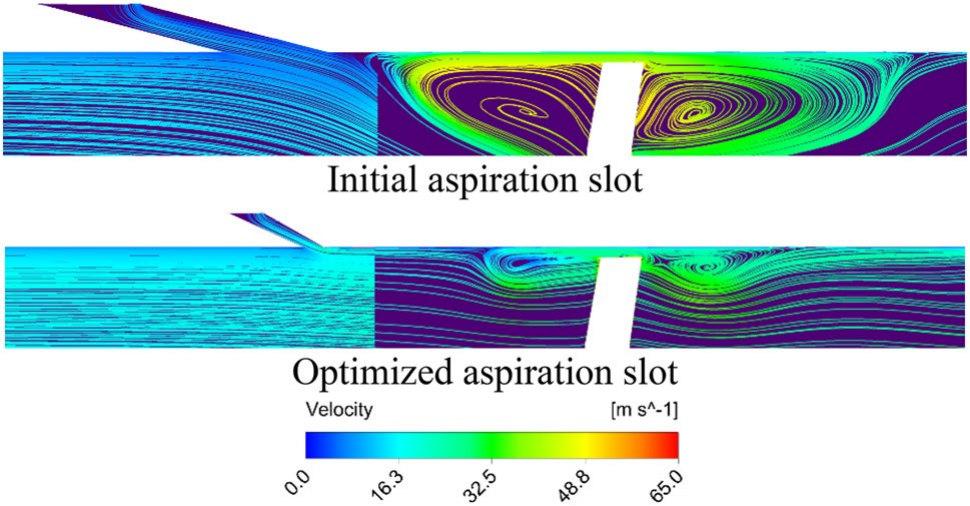
Flow field near compressor casing for initial and optimized aspiration slots at near stall condition.

Flow field near the aspiration slot and blade tip region is shown in Fig. 13 for the initial and optimized cases on the compressor meridional plane. These results belong to the near stall condition. Cross section of the blade leading edge horse-shoe vortex can be recognized in each case. The optimum geometry of the aspiration slot has caused augmentation in the flow momentum entering the blade tip gap, causing enhancement in the flow structure in this region. As a result, the size of the vortical flow in the optimized case has reduced in comparison to the initial case. This beneficial effect can also be observed at the rear region of the blade, where the size of the passage vortex has reduced.

### Quantitaive comparison between different cases

Results of some performance parameters at a cross section of 97% blade span (i.e., very close to the blade tip region) are quantitively shown in Table 4 for three cases of plane, initial and optimized slots. These results belong to the 1.7% tip clearance. Positive effects of utilizing the optimized natural aspiration slot in improving the blade tip leakage flow structure can clearly be deduced from this table (see also Fig. 10). The percentage of enhancement in comparison to the plane case for each parameter is also shown in parentheses. Aspiration through the optimized slot has caused the blockage to the mainstream to reduce. Consequently, the incidence angle decreases, which in turn causes the blade efficiency to increase. In addition, loading factor reduces due to the improved flow structure at the blade tip region of the optimized case (at the same outlet pressure).

**Table 4:** Performance parameters at 97% blade span and 1.7% tip clearance for different cases.

Case study	Efficiency	Total pressure loss coeff	Incidence (deg)	Loading factor
Plane (no treatment)	81.02%	3.06%	$2.9$	0.54
Initial aspiration slot	82.30%	1.90%	$2.5$	0.52
Optimized aspiration slot	84.5% (2.6%)	1.46% (− 14%)	$2.4$(− 3.4%)	0.51 (− 1.8%)

## Conclusion

Findings of present study highlight significance of state-of-the-art natural aspiration in alleviation of undesirable effects of blades tip leakage flow in axial compressors. Through accurate multi-objective optimization process, optimal values for the aspiration slot dimensions (${\theta }_{1}, {\theta }_{2}\text{ and} l$) were determined, demonstrating their pivotal role in enhancing rotor blade row performance. The main conclusions drawn from the current research work can be categorized as follows.

- Natural aspiration through circumferential slot, imposed upstream the blades row in axial compressors, can significantly weaken the blades tip vortex flow strength. Consequently, compressor performance can be improved.
- Careful multi-objective optimization process, driven by cost functions related to average air velocities at the slot outlet (Vs) and the compressor entry (Vc), helped in refining the aspiration slot dimensions.
- Performance evaluation of rotor blades row, particularly examination of pressure rise coefficient variations versus flow coefficient, triggered to find optimum geometry of the circumferential natural aspiration slot. Optimized case exhibited increased pressure rise coefficients and wider stall margins across various blade tip clearance sizes.
- Final results showed that in comparison to the untreated casing, the optimum geometry of the slot causes the total pressure ratio and stall margin to increase by 4.2% and 3%, respectively.
- Detailed insight into flow patterns highlighted the beneficial effects of natural aspiration in weakening the blades tip vortex flow strength.

## Data Availability

The datasets generated and/or analyzed during the current study are not publicly available but are available from the corresponding author on reasonable request.

## List of symbols

$E$: Specific energy of a fluid particle [$\text{J}/\text{kg}$]
*u*: Velocity vector [m/s]
*K*: Specific turbulent kinetic energy [${\text{m}}^{2}/{\text{s}}^{2}$]
*P*: Pressure [$$\text{Pa}$$]
*S*: Entropy [$$\text{J}/\text{kg}.\text{K}$$]
*t*: Time [$$\text{s}$$]
*T*: Temperature [$$\text{K}$$]
*U*: Blade tip speed [m/s]
*C*_*a*_: Axial component of absolute velocity [m/s]
${y}^{+}$: Dimensionless distance of wall first node
*ν*: Kinematic turbulent viscosity [${\text{m}}^{2}/\text{s}$]
$$\rho$$: Density [$$\text{kg}/{\text{m}}^{3}]$$
$$\tau$$: Shear stress [$$\text{Pa}$$]
$\Omega$: Rotational speed [r/min
